# Systematic analysis of the necroptosis index in pan-cancer and classification in discriminating the prognosis and immunotherapy responses of 1716 glioma patients

**DOI:** 10.3389/fphar.2023.1170240

**Published:** 2023-06-07

**Authors:** Shuai Ma, Fang Wang, Qingzhen Liu, Xiaoteng Geng, Zaibin Wang, Menglei Yi, Fan Jiang, Dongtao Zhang, Junzheng Cao, Xiuwei Yan, Jiheng Zhang, Nan Wang, Heng Zhang, Lulu Peng, Zhan Liu, Shaoshan Hu, Shengzhong Tao

**Affiliations:** ^1^ Department of Neurosurgery, The Second Affiliated Hospital of Zhengzhou University, Zhengzhou, China; ^2^ Cancer Center, Department of Neurosurgery, Zhejiang Provincial People’s Hospital, Affiliated People’s Hospital, Hangzhou Medical College, Hangzhou, Zhejiang, China; ^3^ Department of Neurosurgery, The Second Affiliated Hospital of Harbin Medical University, Harbin, China; ^4^ Institute of Psychiatry and Neuroscience, Xinxiang Medical University, Xinxiang, China

**Keywords:** necroptosis index, pancacer, glioma, chemoradiotherapy, immunotherapy

## Abstract

Necroptosis is a programmed form of necrotic cell death that serves as a host gatekeeper for defense against invasion by certain pathogens. Previous studies have uncovered the essential role of necroptosis in tumor progression and implied the potential for novel therapies targeting necroptosis. However, no comprehensive analysis of multi-omics data has been conducted to better understand the relationship between necroptosis and tumor. We developed the necroptosis index (NI) to uncover the effect of necroptosis in most cancers. NI not only correlated with clinical characteristics of multiple tumors, but also could influence drug sensitivity in glioma. Based on necroptosis-related differentially expressed genes, the consensus clustering was used to classify glioma patients into two NI subgroups. Then, we revealed NI subgroup I were more sensitive to immunotherapy, particularly anti-PD1 therapy. This new NI-based classification may have prospective predictive factors for prognosis and guide physicians in prioritizing immunotherapy for potential responders.

## Introduction

Glioma is a common primary intracranial tumor that accounts for approximately 40%–50% of all brain tumors. It is one of the leading causes of cancer-related deaths, prone to chemo-resistance, and one of the main reasons for unsatisfactory treatment outcomes (Huang et al., 2019; Han et al., 2020).

Necroptosis, also known as programmed necrosis, is a regulated form of necrotic cell death mediated by RIP1 and RIP3 kinases. It was initially found to be an alternative to apoptosis following the involvement of receptors in the dead region (Degterev et al., 2005). Although necrosis is widely considered to be a compromise strategy adopted by tumors to create a favorable environment for proliferation and metastasis (DARJAKANDUC et al., 2002), its genetically programmed counterpart, i.e., necroptosis, has been found to exert an inhibitory role in most tumors (Lawlor et al., 2015; Newton, 2015). In some tumor cell lines, two-thirds of the RIPK3 protein levels were decreased, indicating that tumor cells tend to escape necroptosis for survival. Furthermore, low expression of RIPK3 suggests a poorer prognosis for tumor patients (Duan-Wu Zhang et al., 2009; Goodall et al., 2016). Drug-induced necroptosis suppresses tumor growth and decreases tumor metastasis using the accumulated reactive oxygen species (ROS) (Fulda, 2013; Marino et al., 2014; Yang et al., 2018); this may be one reason for the observed relationship between the expression of necroptotic-related genes and patient prognosis.

In the present study, we performed the first comprehensive analysis of necroptosis regulator genes (NRGs) and the necroptosis index (NI) in pan-cancer. The results revealed that necroptosis was related to various cancer hallmarks, mutation, the immune system, stemness, and prognosis. Then, we calculated NI in glioma patients using the single sample gene set enrichment analysis (ssGSEA) algorithm. Subsequently, we obtained necroptosis subgroups and explored differences in genomic variants and the tumor microenvironment between the two necroptosis subgroups through integration analysis to determine the differential efficacy of immunotherapy and chemotherapy. These findings discovered the important role of necroptosis in tumors and contributed to further study of necroptosis-related molecular mechanisms. In future, this could assist physicians and glioma patients to individualize survival prediction and provide better treatment choices based on NI classification.

## Methods

### Data extraction

RNA_seq, copy number alteration data, and clinical characteristic of pan-cancer were gathered from UCSC Xena Browser (https://xenabrowser.net/datapages/). RNA_seq (FPKM) and clinical information for 698 and 1,018 glioma samples were obtained from the TCGA and CGGA databases. Mutation data of low-grade glioma (LGG) and Glioblastoma (GBM) were download from TCGA.

### Recognizing necroptosis regulators

In a recent study from 2016, Tania Love Aaes et al. discovered that RIPK1, RIPK3, FADD, and MLKL were the key factors for necroptosis (Aaes et al., 2016). In 2021, Han-Hee Park et al. found that RIPK1, RIPK3, TNF, and MLKL are also proposed to be key molecules in necroptosis (Park et al., 2021). Kim Newton et al. identified TLR3, FASLG, and FAS as key factors in necroptosis in 2016 (Newton and Manning, 2016). Finally, we combined with MSigDB Team (GOBP_NECROPTOTIC_SIGNALING_PATHWAY) (https://www.gsea-msigdb.org/gsea/msigdb/) to obtain these eight Necroptosis-related genes (FADD, TNF, FASLG, MLKL, TLR3, RIPK1, FAS, and RIPK3) (Linkermann and Green, 2014).

### Establishing the necroptosis index

The necroptosis index to represent the necroptosis level was established based on the expression data for genes of necroptosis key genes including positive components of FADD, TNF, FASLG, MLKL, TLR3, RIPK1, FAS, and RIPK3. The enrichment score of these genes that regulated necroptosis was calculated using ssGSEA in the R package ‘GSVA’.

### Gene set enrichment analysis

To identify the pathways associated with necroptosis, the samples of each tumor type were divided into two groups according to the NI, consisting of the top 30% and bottom 30%. Then, the gene set enrichment analysis was performed.

### RNA extraction, RT-PCR, and qRT -PCR

Tissue RNA isolation total RNA was extracted from eight Glioblastoma (GBM) and control brain tissue samples. Human specimens were approved by the Ethics Committee of the Second Hospital of Harbin Medical University. TRIzol reagent (Invitrogen) was used to extract total RNA from the cells and tissue specimens. Primers for eight genes were synthesized from Tsingke Biotech (Beijing, China). PrimeScript RT reagent kit with gDNA Eraser (Takara Bio, Inc.) was used to prepare cDNA, and SYBR Green II mixture (Takara Bio, Inc.) was used for RT-qPCR. Calculation of target mRNA levels was based on the CT method and normalization to human ACTB expression. The original PCR data and analysis process of these 16 tissue samples are presented in Supplementary Table S7.

### Identification NI subgroups

Consensual clustering uses the k-means algorithm to identify specific NI subgroups associated with the expression of NRGs. Number and stability of clusters were decided by the consensus clustering algorithm using the “ConsensuClusterPlus” package. We conducted the experiment with 1,000 iterations to ensure the robustness of our categorizations (Wilkerson and Hayes, 2010; Zhang et al., 2020).

### Mutation data in NI subgroups

We detected the SNVs, SNPs, and INDELs using the software VarScan2 (Koboldt et al., 2012a). The co-occurrence and mutually exclusive mutations were identified using theCoMEt algorithm (Leiserson et al., 2015). Mutation data were analyzed in two groups and visualized using the “maftools” R package (Koboldt et al., 2012b).

### Differential expression genes analysis of NI subgroups

To test genes differentially expressed between NI subgroups, gene expression data for glioma RNA_seq were downloaded from TCGA. Then, the fold change and adjusted *p*-value were calculated by the limma package (Ritchie et al., 2015). We defined genes with an adjusted *p*-value less than 0.05 and fold change<|2| as the differential expression genes (DEGs).

### Clinical features analysis

The R package “survival” was used to assess the prognosis potential of the NRGs and necroptosis index among tumors. For survival analysis, the expression threshold was exhaustively tested and the one with most significant *p*-value was considered the best cut-off.

### Immune infiltration

The ssGSEA was applied to detect the infiltrating scores of 28 immune cells. Feature gene panels for each immune cell type were obtained from a recent publication (Charoentong et al., 2017). The relative abundance of each immune cell type was represented by an enrichment score in ssGSEA analysis. The ssGSEA score was normalized to unity distribution, for which zero is the minimal and one is the maximal score for each immune cell type. The bio-similarity of the immune cell filtration was estimated by multi-dimensional scaling (MDS) and a Gaussian fitting model.

### WGCNA

The key genes in 4,645 DEGs were identified by applying WGCNA. First, we constructed the adjacency matrix according to the connectivity of the best β values in order to make gene distributions conform to the scale-free network and transformed the adjacency matrix to topology overlap matrix (TOM). Next, we used the heterogeneity among genes to aggregate the genes for the TOM. Finally, the identified TOMs were defined as components and dynamical tree cutting algorithm was used for stratified clustering to identify modules with minimum module size of 25 (Langfelder and Horvath, 2008; Yi et al., 2020).

### Significance of the NI subgroups in chemotherapeutic sensitivity

An algorithm developed by Geeleheret et al. (Paul Geeleher and Huang, 2014) and the “pRRophetic” package (Geeleher et al., 2014) were used by the TCGA project to compute the IC50 of commonly used chemotherapeutic agents in order to evaluate the clinical efficacy of NI subgroups. The AJCC guidelines recommend 30 common antineoplastic agents for cancer treatment, such as Imatinib, Adriamycin, Cisplatin, and Vinblastine. The distinction in IC50 of commonly used drugs in two NI subgroups was assessed by the Wilcoxon test.

### Statistical analysis

All statistical analyses were executed with R version 4.0.5 (Yoshihara et al., 2013). Adjustment for multiple testing was used to compare differences in immune and mutation status between NI subgroups. *p* < 0.05 was regarded as statistically significant.

## Results

### Aberrant expression of necroptosis index in cancers

**FIGURE 1:**
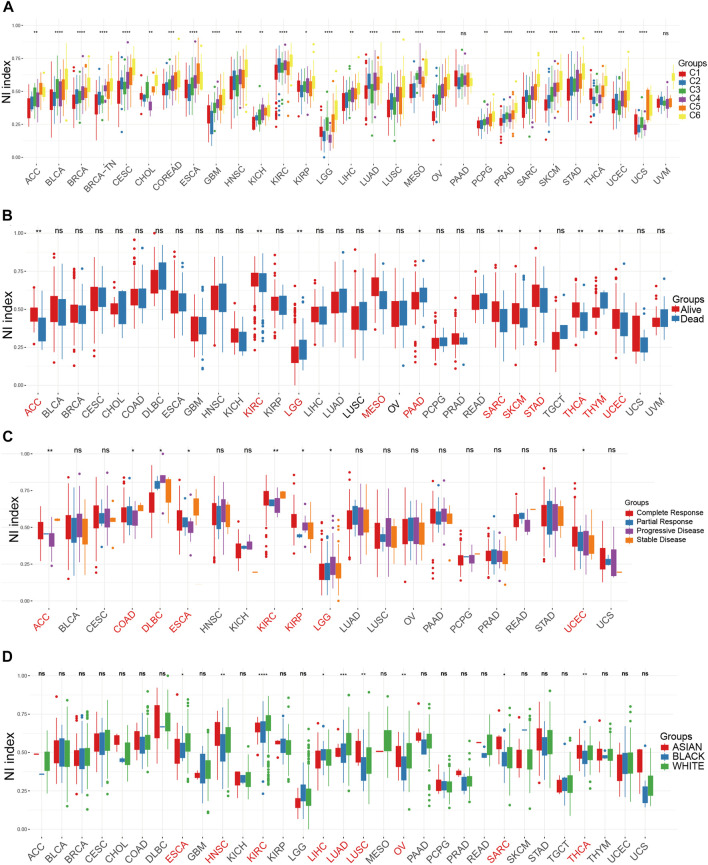
The relationship between clinical factors and NI. **(A–D)** The differential expression of NI among Immunophenotype **(A)**, prognostic results **(B)**, treatment response **(C)**, and race **(D)** in pan-cancer.

In the present study, the eight genes extracted from MSigDB that play crucial roles in the regulation of necroptosis were identified as NRGs, and included FADD, TNF, FASLG, MLKL, TLR3, RIPK1, FAS, and RIPK3. In order to further understand the importance of necroptosis in tumor progression and explore the factors or biological mechanisms relevant to necroptosis, NI was modeled based on the positive core group component enrichment fraction minus the negative core group component enrichment fraction calculated by ssGSEA (Supplementary Table S1). First, we studied the relationship between NI and molecular features. NI were clearly distinguished in all tumor types according to immunophenotype from a previous article (Figure 1A). We found that the NI of C4, C5, and C6 were significantly higher than those of C1, C2, and C3. We know C3 had the best prognosis, while C2 and C1 had less favorable outcomes. In our study, C4 and C6 had poor clinical outcomes. Thus, the NI in most tumors could respond to the immune status and prognosis of patients. Of all the tumors, NI in 11 tumors showed remarkable distinctions in survival status (Figure 1B), these were ACC, KIRC, LGG, MESO, PAAD, SARC, SKCM, STAD, THCA, THYM, and UCEC. NI in eight tumors revealed markedly different between-treatment results (Figure 1C): ACC, COAD, DLBC, ESCA, KIRC, KIRP, LGG, and UCEC. NI in nine tumors indicated a significant difference in race, which were ESCA, HNSC, KIRC, LIHC, LUAD, LUSC, OV, SARC, and THCA (Figure 1D). The NI of women was higher than men in HNSC, LUAD, and STAD but lower in LIHC (Supplementary Figure S1G). Finally, we performed cox model of NI in pan-cancer. The result showed that ACC, GBM, LGG, LIHC, MESO, PAAD, SARC, SKCM, STAD, THCA, and THYM were obviously meaningful and had prognostic value (Supplementary Figure S1F).

### Association between necroptosis and genetic alterations and pathways in tumors

**FIGURE 2:**
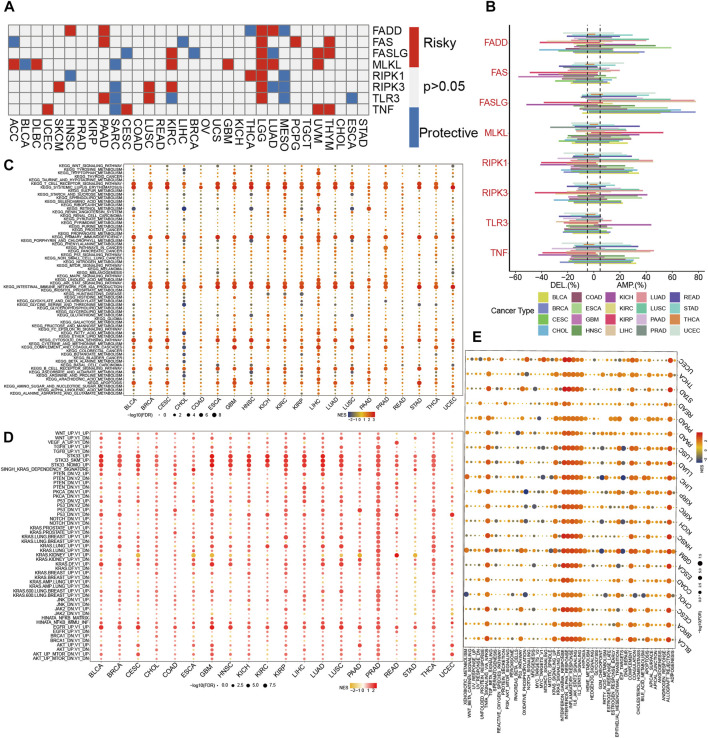
The dysregulation of necroptosis regulators. **(A)**The correlation between expression of necroptosis regulators and patient survival in pan-cancer. **(B)** Histogram displays the rate of somatic copy number alterations for NRGs in pan-cancer. **(C–E)** Enrichment analysis for metabolism pathway **(C)**, cancer signaling **(D)**, and hallmark gene sets **(E)** between high- and low-NI tumor tissues.

**FIGURE 3:**
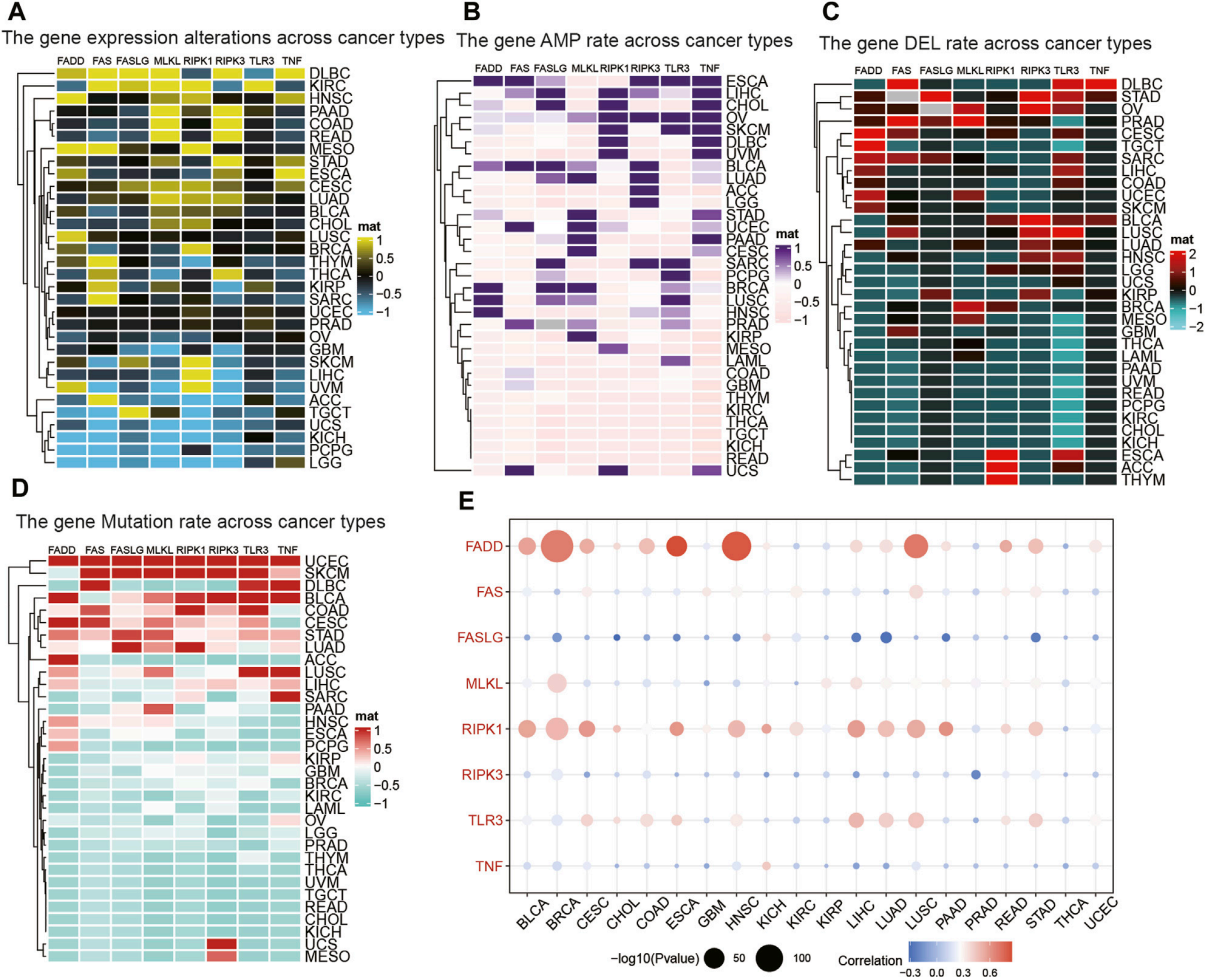
The relationship between AMP, DEL, mutation rate, and their expression values of NRGs. **(A)** The expression of NRGs in pan-cancer. **(B)** The AMP of NRGs in pan-cancer. **(C)** The DEL of NRGs in pan-cancer. **(D)** The mutation rate of NRGs in pan-cancer. **(E)** The correlation between the expression and CNV in NRGs.

We found that all of the NRGs were related to the prognosis of patients in most tumor types, except PRAD, KIRP, READ, OV, UCS, KICH, TGCT, and STAD (Figure 2A). This finding revealed that NRGs were associated with the prognosis of most tumor patients. To further explore the mutation profile of NRGs in tumors, the proportion of somatic copy number alterations (SCNA) was detected, and the results demonstrated that SCNA possess a high rate (more than 5%) in most cancers (Figure 2B), but the SCNA frequencies of NRGs were low in THCA. Furthermore, NRGs displayed different SCNA profiles. For instance, FADD, FASLG, RIPK1, RIPK3, and TNF were more prone to copy number gain than loss in pan-cancer, but FAS and TLR3 displayed the reverse tendency. To further clarify the association between the NI and pathways, we applied GSEA to investigate the related cellular signaling of necroptosis in tumors based on the RNA_seq of tumors with the top and bottom 30% of NI. It was observed that metabolism-related pathways in KEGG were usually enriched in tumors with lower NI; pathways frequently enriched (>7 cancers) are presented in Figure 2C. For example, T_cell_receptor_signaling_pathway, Systemic lupus erythematosus, Primary_immunodeficiency, JAK_STAT_SIGNALING_pathway, and Intestinal_immune_network_for_iga_production were enriched in the high-NI group in most cancers. Purine_metabolism, Mtor_signaling_pathway, and Galactose_metabolism were also significantly correlated with low-NI in all these cancer types, which indicated that necroptosis was negatively related to these metabolism-related pathways (Figure 2C). Furthermore, the relationship between NI and cancer hallmarks were also analyzed, and the results showed that 12 hallmarks were frequently significantly correlated with NI (Figure 2E). For example, INTERFERON_GAMMA_RESPONSE, INTERFERON_ALPHA_RESPONSE, INTERFERON_ALPHA_RESPONSE, and IL6_JAK_STAT3_SIGNALING were enriched in the high-NI group. This indicated that necroptosis was positively related to these cancer hallmarks. Finally, most oncogenic signatures were also significantly negative with low-NI in pan-cancer. These results are consistent with necroptosis activating tumor immunity and inhibiting tumor growth (Figure 2D). We analyzed the relationship between amplification (AMP), deletion (DEL), mutation rate, and their expression values of these eight genes in Figures 3A–D. Figure 3A showed the expression of eight genes in pan-cancer, Figure 3B displayed the AMP of eight genes in pan-cancer, Figure 3C revealed the DEL of eight genes in pan-cancer, and Figure 3D showed the mutation rate of eight genes in tumor. From the above results, we found that the expression of NRGs with CNV AMP was significantly higher in cancer cells (e.g., FADD, FASLG, RIPK3, and TNF), while the expression of NRGs with CNV DEL was significantly lower (e.g., RIPK1 and TNF). The expression values of FADD, MLKL, RIPK1, and TLR3 were positively correlated with their CNV, while FASLG, RIPK3, and TNF were the opposite (Figure 3E). In conclusion, these findings implied that crosstalk among the NRGs plays a crucial role in the development and progression of most cancer types.

### The efficacy of the NI across tumor types

**FIGURE 4:**
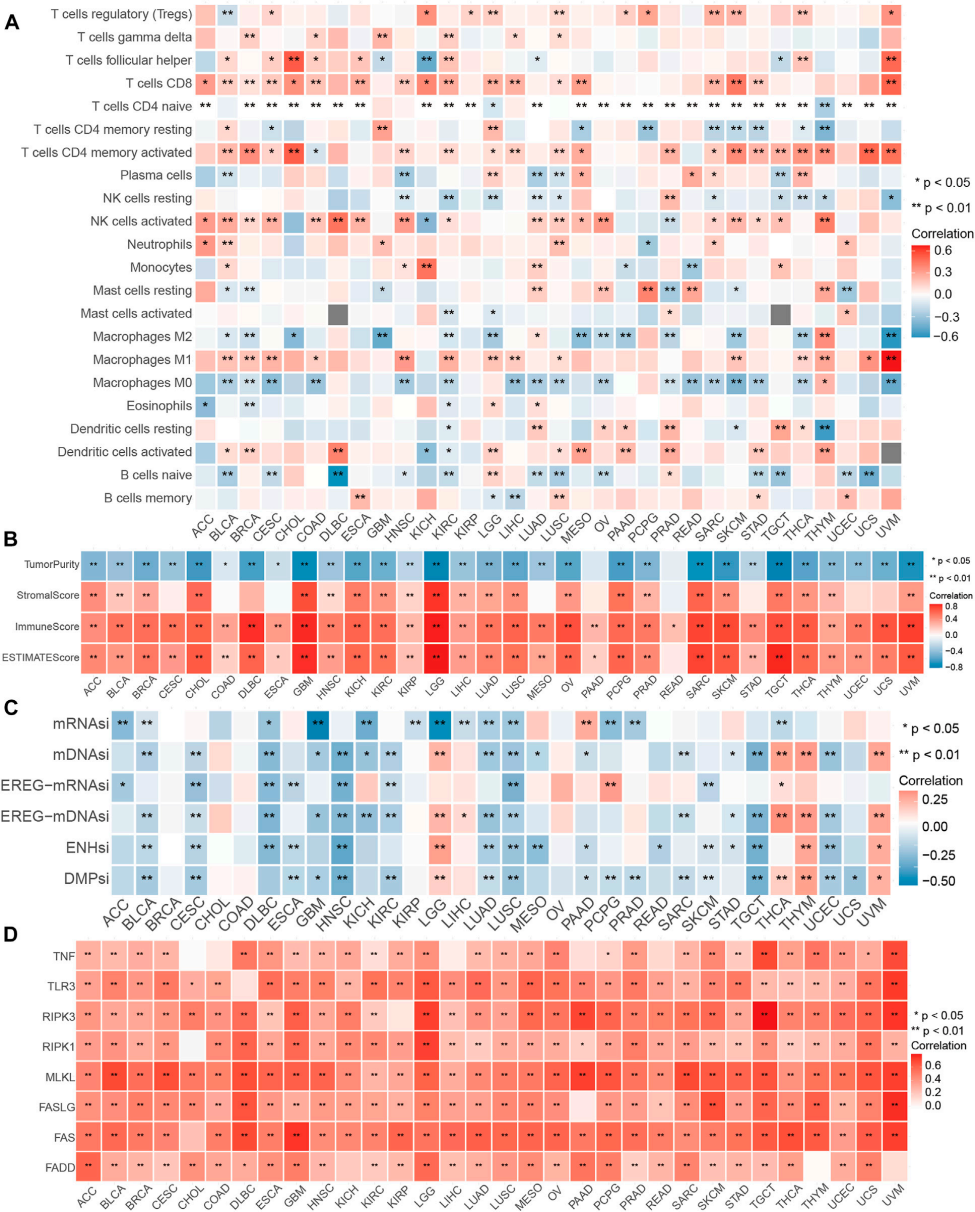
Performance of NI in pan-cancers. **(A)** Relationship between the immune cells and NI in pan-cancer. **(B)** Correlations between the NI and ESTIMATE score in pan-cancer. **(C)** Association between the NI and stemness indices in pan-cancer. **(D)** Correlations between the NI and NRG in pan-cancer.

Considering the solid relationship between necroptosis and signaling pathways described above, we further investigated the potency of the NI in different cancer types. We calculated the relationship between the 28 kinds of immune cells and NI and discovered a significant correlation among all tumor types. The percentage of Tregs, T cells follicular helper, T cells CD8, T cells CD4 memory active, NK cells activated, Macrophages M1, and naive B cells were relevant to the NI of most tumor types (Figure 4A; Supplementary Table S2). Interestingly, these cells were antitumor types, suggesting, to some extent, that the NI facilitates tumor immunity. Furthermore, we found a significant correlation between NI and stem cell indices for cancer types, except for BRCA, CHOL, COAD, and OV (Figure 4C; Supplementary Table S3). Meanwhile, we also observed an significant association between the Estimate score and the NI in all tumors (Figure 4B; Supplementary Table S4). We investigated the relationship of NRGs with NI and found that NRGs were significantly positively correlated with NI (Figure 4D; Supplementary Table S5). Finally, we found that NI was significantly correlated with the prognosis of patients in nine tumors: STAD, MESO, GBM, SKCM, LIHC, LGG, ACC, KIRC, and THYM. This result revealed that NI could significantly affect the prognosis of tumor patients (Supplementary Figures S1A–C; Supplementary Table S6). Significant indicators reflecting the reaction to immune checkpoint therapy can be broadly classified into two types: microsatellite instability (MSI) or TMB, and inflammatory infiltrating. The radar plots indicated significant correlation between NI and TMB in 16 tumors (Supplementary Figure S1D). Subsequently, we examined the association between NI and MSI and discovered that COAD displayed the largest positive relevance. These findings may indicate excessive T cell infiltration in DLBC (Supplementary Figure S1E). By analyzing the two immune-related indicators, the correlation between the indicators and reaction was reversed in some tumors. This phenomenon might be related to the heterogeneity of immune infiltration among cancers. For instance, PAAD had a highly positive relevance with TMB and a negative relevance with MSI values, which may be associated with the nontypical immunogenicity of PAAD. The results of these pan-cancer analyses demonstrated the immunological, mutational, and prognostic value of NI in a variety of tumors.

### The landscape of NRGs in glioma

In Figure 2A, we found a clear effect of NRGs on the prognosis of glioma. Thus, we further explored the role of NRGs in glioma and found that the alterations of all NRGs were common and mainly focused on copy number amplification (Supplementary Figure S2B). We identified the alterations in CNV characteristics of all the NRGs on the chromosome. These findings revealed that the CNV state of all NRGs is associated with the proliferation and development of glioma. We further investigated the relevance of the NRGs and found that RIPK3 was significantly correlated with other genes, with the highest coefficient of correlation (0.8) between RIPK3 and MLKL (Supplementary Figure S2C). In addition, we studied the correlation between the expression patterns of NRGs and molecular features. Of the NRGs, seven genes showed a significant difference between normal tissues and glioma, while RIPK3 did not (Supplementary Figure S2D). The NRGs were distinct in groups classified according to IDHmut subtypes except RIPK3 (Supplementary Figure S2E). Of the NRGs, seven revealed remarkable distinctions among WHO classification, except TNF (Supplementary Figure S2F). The mutation frequency of NRGs in the 660 samples was 1.52%, and were mostly missense mutations. MLKL exhibited the greatest mutation rate, followed by other NRGs which did not show any mutations in glioma samples (Supplementary Figure S2G). We performed PCR validation of NRGs and found that all genes except RIPK3 were significantly different between tumor and normal tissues (Supplementary Figure S2H; Supplementary Table S7). This result is consistent with the expression of NRGs in the TCGA, indicating the stability of the expression of NRGs. As a result of these findings, we found significant differences in the expression of NRGs, which may play critical roles in glioma development.

### Associations between the NI and clinical features

**FIGURE 5:**
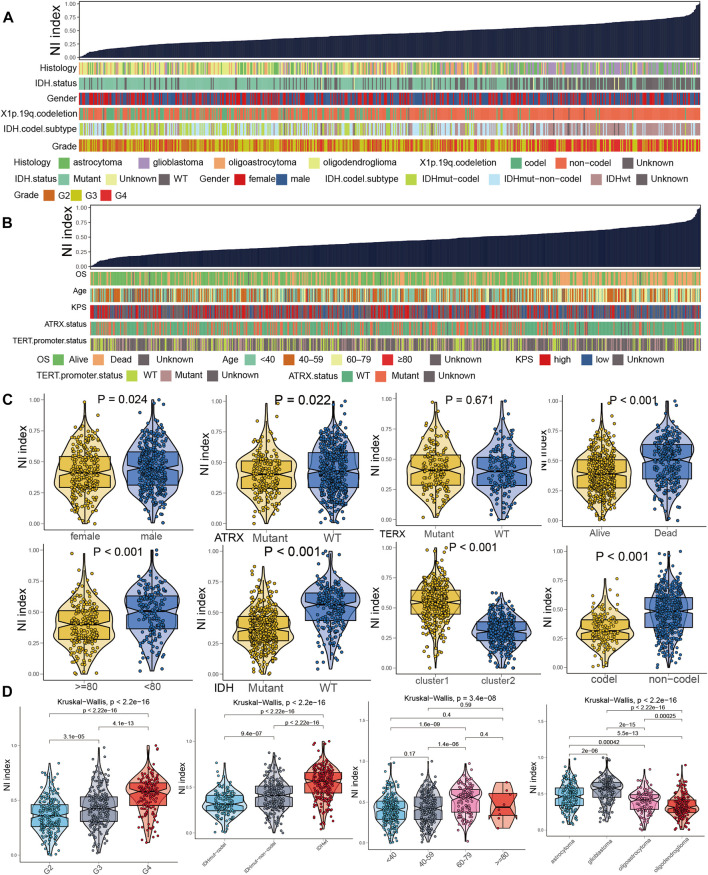
Clinical and molecular characteristics of glioma associated with NI. **(A,B)** A comprehensive analysis of the relationship between NI and clinicopathological characteristics of patients. **(C)** Violin plots of NI in glioma patients, stratified by gender, age, ATRX mutation, IDH mutation, TERX mutation, OS status, and 1p19q codel. **(D)** Violin plots of NI in glioma patients, stratified by grades, IDH mutation, ages, and histological classification.

By applying the ssGSEA algorithm, the NI was computed according to the RNA sequence of 698 glioma samples and then ranked from lowest to highest to investigate the association between molecular classification and clinical characteristics (Figures 5A, B). As displayed in Figure 5C, the NI of male patients was significantly higher than female patients, and the NI in dead patients was obviously higher than in alive patients, which suggests that NI could reflect the prognosis of glioma patients. NI was significantly higher in patients older than 80 years than in those younger than 80 years. Patients with 1p19q codes had significantly lower NI than those with non-codes. The patients were significantly higher in IDH-mutant and ATRX-mutant samples than in wild-type samples. As shown in Figure 5D, NI in patients increased significantly with the increase of tumor grade, all of which were statistically significant. Patients aged 60–79 years had significantly higher NI than 40–59 and <40 patients. Meanwhile, there was no significant difference between groups in NI values in the ≥80 group. Among the WHO classification, the GBM had the highest NI, followed by astrocytoma, oligoastrocytoma, and oligodendroglioma. Furthermore, patients with IDHwt subtype demonstrated higher NI than IDHmut-non-codel and IDHmut-codel. These results suggest that NI is positively correlated with the malignancy and age of glioma and can reflect the condition and prognosis of glioma patients.

### Associations between the NI and immune microenvironment

**FIGURE 6:**
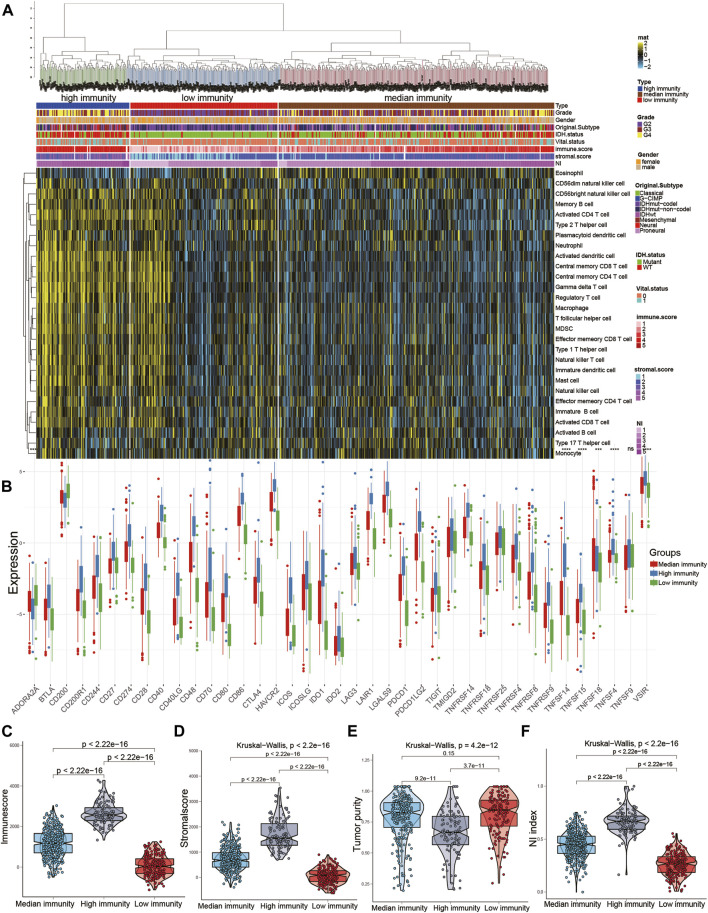
Relationship between NI and immune subgroups of gliomas. **(A)** The immune subgroups of glioma were classified based on the overall immune activity of glioma. **(B)** Quantitative analysis of the proportion of immune checkpoint in three immune subtypes. **(C–F)** Quantitative analysis of NI and ESTIMATE score in three immune subtypes.

The ssGSEA scores of 29 immune cells were sorted into three immune subgroups using hierarchical clustering. 229 cases (32.8%) had the highest enrichment scores and were considered as high immune group and “hot immune” tumors. 203 cases (29%) had the lowest enrichment scores and were considered as low immune group and “cold immune” tumors. 353 cases (50.5%) had the medium enrichment scores and were considered as medium immune group and “altered immune” tumors, indicating the potential to conversion to cold or hot tumors (Figures 6A,B). The immune checkpoints in high immunity group was higher than other groups. Then, we further investigated the relationship between NI and immunity. The NI was significantly positively correlated with the immune score and stromal score, indicating that, as the NI of glioma increased, the level of infiltration of immune cells and stromal cells increased (Supplementary Figures S3C, E). However, significant negative correlation between NI and tumor purity was observed (Supplementary Figure S3D). Then, the enrichment scores of 22 kinds of immune cells and immune checkpoints were quantified by the ggplot (Supplementary Figures S3A, B). The results revealed the high immune group had the highest scores of immune cells and immune checkpoints, followed by medium and low immune groups. In addition, the immune score and stromal score were both the highest in high immune subgroups, indicating high enrichment scores of stromal cells and immune cells, followed by medium and low immune subgroups (Figures 6C, D). By contrast, tumor purity increases gradually from high to low immune subgroups, and NI gradually decreases. Moreover, the NI in high immunity was higher than other two groups (Figures 6E, F). From the above findings, we suggest the NI has significant association with the immune status of glioma.

### Identification of two NI subgroups with different OS and clinical characteristics

We performed DEGs between high and low groups of NI with limma package and obtained 4,645 DEGs and performed univariate and multivariate cox to obtain 125 DEGs. Then, we used consensus clustering to discover a new classification of glioma on the basis of the RNA sequence of 125 necroptosis-related DEGs. Based on the CDF curve, the consensus heatmap, and the PAC algorithm, the best number of clusters was identified as two (k = 2) (Supplementary Figures S4A–C). We also displayed the clusters (k = 2–6) in Supplementary Figures S5A–F. Therefore, glioma patients were classified into two subgroups, which were termed NI subgroup I (359 patients, 51.4%) and NI subgroup II (339 patients, 48.6%). Glioma patients in the NI subgroup II presented superior OS than those in the NI subgroup I in the TCGA and CGGA (Supplementary Figures S4I, J). Then, we found a significantly lower number of high-grade patients in the NI subgroup II than in the NI subgroup I, as well as a higher proportion of deaths, a higher number of patients with IDH mutation, a higher number of patients with 1p19q codel, a higher percentage of MGMT methylation, and a higher percentage of ATRX (Supplementary Figures S3F, S4D–H). For the original subgroup and methylation subgroup, the proportions of idhmuton-codel and IDHmutnon-codel in NI subgroup II were higher than other types, and the proportions of codel and G-CIMP-high in NI subgroup II were higher than other types (Supplementary Figures S3G, H).

### Identification of the characteristic of NI subgroups with immunity

**FIGURE 7:**
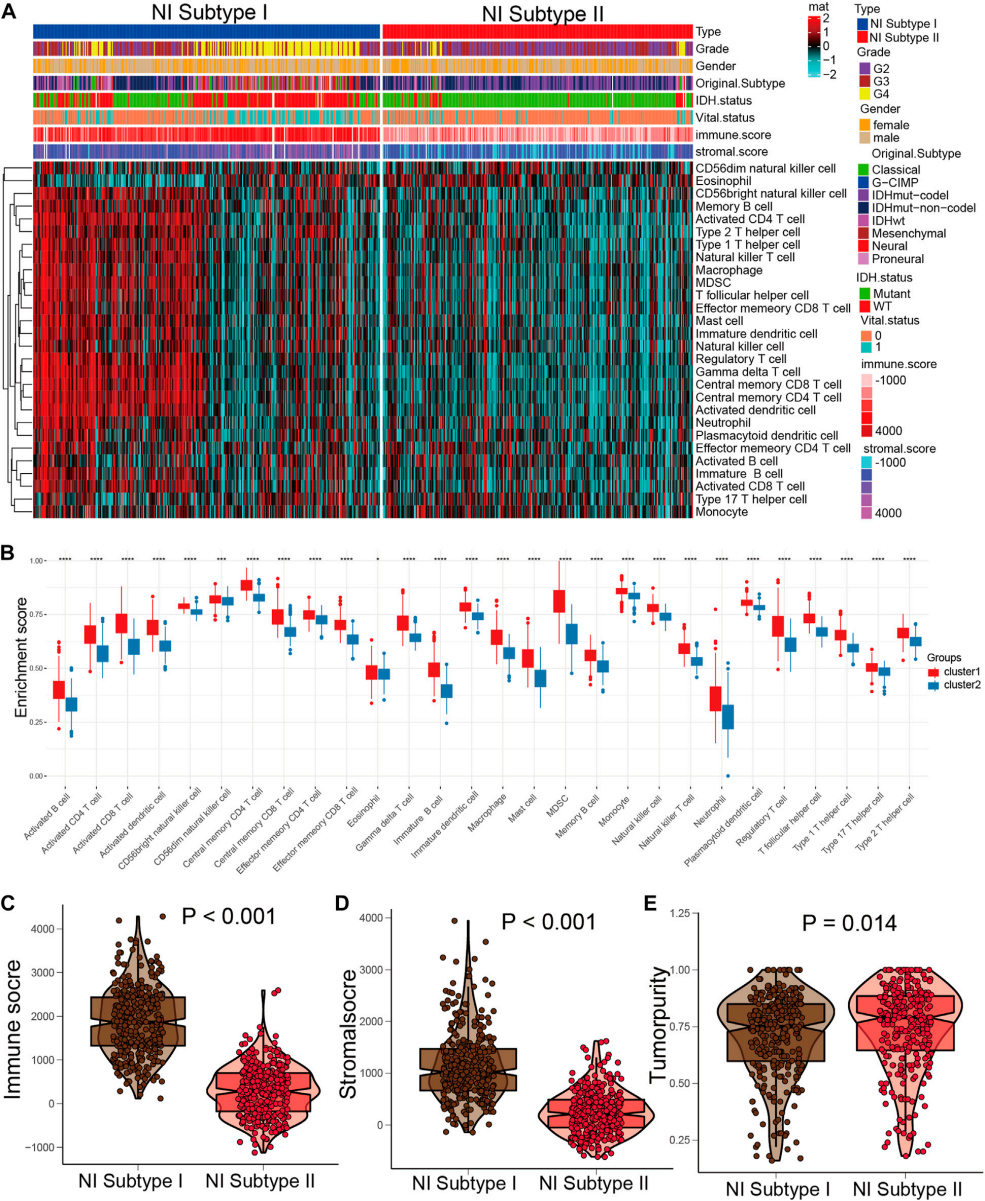
Distinct immune patterns of two NI subgroups. **(A)** Quantitative analysis of ESTIMATE score between two NI subgroups. **(B)** Quantitative analysis of the proportion of immune cells in two subgroups. **(C–E)** The expression levels of ESTIMATE score in NI subgroup I and II.

In the previous studies, depleted immune and active immune subgroups differed significantly in B cells, cytolytic activity, and M1/M2 macrophages, but not in cytotoxic cells, CD8T cells, and T cells. Immune-active subtypes are strongly associated with immune-active pathways and gene sets, and immune-depleted subgroups are closely related to tumor-promoting signals that restrain host immune response, like activation of the Wnt/TGFβ1 signaling pathway (Chen et al., 2019). To investigate the correlation between NI subgroups and tumor immunity, we investigated the abundance of immune cells’ infiltration in NI subgroups and found that activated B cells, activated CD4 T cells, and activated CD8 T cells were significantly higher in NI subgroups I than in NI subgroups II (Figures 7A, B). Immune score and Stromal score in NI subgroup I are significantly higher than NI subgroup II, while tumor purity is the reverse (Figures 7C–E). These results suggest that NI subgroups were closely associated with tumor immunity.

### Identification of the characteristic of NI subgroups with mutation

Prior research confirmed the potential role of mutation in regulating tumor immunity (Rooney et al., 2015; Thorsson et al., 2018). Therefore, we performed CNA and somatic mutation profiling to investigate the different mutation status in the two NI subgroups. As displayed in Supplementary Figures S6A, B, NI subgroup I had a higher mutation rate (90.88%) than NI subgroup II (95.02%) (Supplementary Figures S6A, B). The IDH1 mutation rate was higher in NI subgroup II (82%) than NI subgroup I (36%), IDH1 mutation dramatically indicated the outcome of glioma patients, so the distinction in IDH1 mutation between two cluster subgroups may contribute to the prognosis of glioma. Moreover, we examined the landscape of co-occurrence using the top 25 mutation genes with the comet algorithm. Twelve pair cases (EGFR-TP53, MUC16-TP53, PTEN-IDH1, EGFR-IDH1, NF1-IDH1, PTEN-ATRX, EGFR-ATRX, ATRX-CIC, ATRX-FUBP1, ATRX-PIK3CA, TP53-CIC, TP53-FUBP1, TP53-NOTCH1, TP53-ZBTB20, IDH1-IDH2, IDH-EGFR, and IDH1-PTEN) were compared with prevalent mutually exclusive mutations, indicating that they may have a superfluous impact on the common pathway and a selected advantage of retaining the mutation copy between them (Supplementary Figure S6E). After detecting RNA sequence alterations in both subgroups, we further explored genomic-level distinctions between the two NI subgroups. Somatic mutation, including single nucleotide variants, insertions, single nucleotide polymorphisms (SNP), and deletions, were computed and visualized applying the “maftools” package. The SNPs and Total in NI subgroup I were also exceeded by those in NI subgroup II (Supplementary Figure S6H). More interesting is that several genes had distinct mutation rates between the two cohorts. In terms of outcomes, the top 10 genes were shown in Supplementary Figure S6I. Furthermore, IDH1 is another classic example demonstrating the distinct mutation sites between two cohorts (Supplementary Figures S6C, D) and the plausible chain reaction of the variance in prognostic effect. Eventually, we evaluated driver genes for the two NI subgroups, and the findings indicated that the dominant driver genes of NI subgroup I were PLCH2 and IDH1, meanwhile, the driver genes of NI subgroup II subgroup were IDH1 and IDH2 (Supplementary Figure S6G). Moreover, the samples in the NI subgroup I have remarkably higher enrichment scores of variant allele fractions than those in the NI subgroup II (Supplementary Figure S6F), which had been thought to be linked to cancer progression and worse prognosis.

### Comparison of DEGs in the two NI subgroups

Given the prognostic distinctions between the high and low subgroups, we investigated DEGs between the two subgroups. 4,645 DEGs were identified: 2,219 genes were upregulated and 2,426 genes downregulated in the high NI group. The most upregulated genes were BRSK2, MAST1, CTIF, JPH4, ADGRL1, SCAMP5, SLC25A27, CRIPAP1, USP11, and RUNDC3A, while the most downregulated genes were ARHGDIB, TMEM109, LAT2, HLA-DMA, SPI1, SASH3, LYN, CXCL16, NAGA, and FERMT3 (Supplementary Figure S4K).

### Construction and validation of the NI subgroups predictor

The samples were aggregated in a scale-free network using WGCNA algorithm, and 4,645 DEGs co-expression modules were found (Supplementary Figure S7C). The obtained topology matrix was clustered based on the β value to the proximity and topology matrix and based on the differences between genes. The hierarchical clustering method was applied to generate the gene dendrogram. An assignment of modules identified by dynamic cutting tree is shown in the colorful rows at the bottom of the tree diagram (Supplementary Figure S7A). Closed modules were merged into new modules, and the characteristic genes were calculated for each module. As shown in Supplementary Figure S7B, a total of 4,645 DEGs were divided into 24 modules. Since the ME yellow module (GS > 0.5, MM > 0.8) had the highest correlation with NI, we performed a NI subgroups predictor screen for genes in this module. The yellow module obtained 125 genes significantly associated with NI; we then performed multi-cox and lasso regression on 125 genes, and finally obtained 10 hub genes: C3, DOK3, FCER1G, FCGR2A, FCGR3A, GNA15, IL10RA, LRRC25, RGS19, and WAS (Supplementary Figure S7D). We performed model construction based on risk coefficients for these 10 hub genes and found that patients in the low-risk group had significantly higher survival than the high-risk group in the TCGA and CGGA cohorts (Supplementary Figures S7E, F). Finally, timeROC analysis in the TCGA cohort showed that the AUC is greater than 0.79 at 1, 3, 5, and 7 years and greater than 0.74 in the TCGA and CGGA cohorts (Supplementary Figures S6G, H).

### Clinical application of NI subgroup

Different NI subgroups should contribute to the clinical treatment of glioma. Therefore, we calculated the sensitivity of the two NI subgroups for 30 anticancer drugs to identify appropriate agents for glioma treatment by using the pRRophetic algorithm. 24 chemotherapeutic drugs had obviously distinct IC50 in the two NI subgroups (Supplementary Figure S8A; Supplementary Table S8). Then, subclass mapping algorithm was employed to forecast the outcome for ICI therapy, containing CTLA4 and PD1 inhibitors. We discovered that the NI subgroup I has a better outcome in anti-PD1 therapy (Supplementary Figure S8B; Supplementary Table S9).

## Discussion

Necroptosis is a specific form of cellular necrotic death mediated mainly by MLKL, RIP1, and RIP3 (Gong et al., 2019; Martens et al., 2021). In tumor therapy, necroptosis can be used as a programmed death modality to avoid apoptosis resistance and enhance anti-tumor immunity (Frank and Vince, 2018; Sprooten et al., 2020). Nevertheless, there is an absence of comprehensive studies on necroptosis and NRGs in pan-cancer. In present research, we utilized multiomics and clinical features from TCGA to reveal overall alterations of NRGs at genetic, transcriptional, and epigenetic levels. We also processed expression data using ssGSEA to construct NI to feature necroptosis and determine which genes and non-gene factors are associated with NI. Distinct molecular types affect the NI in most cancers, implying that distinct molecular subgroups responding to therapy may be associated with necroptosis.

It is unclear how necroptosis mediates glioma proliferation, but the association we discovered between NI and cancer features could increase the knowledge on necroptosis. GSEA revealed that the degree of NI is strongly correlated with tumor-associated oncogenic signaling pathway, cancer hallmarks, and metabolism-related pathways in pan-cancer. NRGs can act as both oncogene and tumor suppressor, and the NI plays the role of a protection or risk factor in different tumors. We also discovered that some clinical features affected necroptosis, like therapeutic response, survival status, and immune phenotype. NI also differs between genders in some tumors, including HNSC, LUAD, STAD, and LIHC, and between races in HNSC, LUAD, ESCA, LIHC, LUSC, KIRC, OV, THCA, SARC, and ESCA, implying the need to consider gender and race when considering necroptosis as a treatment strategy. We also observed that superior clinical outcome or status was also associated with higher NI in several cancer types, which further confirmed the double effect of necroptosis. Therefore, a distinct method of modulating the necroptosis of tumor cells may be beneficial to patients and enhance prognosis.

In addition, we found that most NRGs and NI were significantly associated with the prognosis of GBM and LGG in Figure 2A, and we went on to explore the effect played by NRGs in glioma. Then, we thoroughly analyzed the relationship between the necroptosis of glioma and the response of chemotherapy and immunotherapy, and developed the method to differentiate subtypes based on necroptosis. First, we applied the ssGSEA to construct the NI in 1716 glioma samples from a public database. We classified the glioma patients into two subgroups on the basis of their NI and compared their clinical characteristics to identify the relationship between the NI subgroups and clinical characteristics. ICI therapy, especially anti-PD1 therapy, can obtain better treatment results in NI subgroup I, as predicted by the subclass mapping algorithm, while chemotherapeutic agents are effective, as predicted by the pRRophetic algorithm. Furthermore, to distinguish clinical value between these two NI subgroups, we identified the 10 crucial necroptosis subgroup-related DEGs by lasso, univariate, and multivariate cox, and regarded them as predictors of necroptosis subgroup. The 10 genes were C3, DOK3, FCER1G, FCGR2A, FCGR3A, GNA15, IL10RA, LRRC25, RGS19, and WAS.

Although the central nervous system is a relatively specific immune region, immunotherapy has been extensively investigated for glioma in recent years, mainly containing cellular immunotherapy, ICI, and anti-tumor vaccines. Nevertheless, the efficacy of these phase III clinical trials in GBM have been unsatisfactory compared with other tumors (Weller et al., 2017; Reardon et al., 2020). There are numerous parameters that influence the efficacy of GBM immunotherapy. In the case of PD-L1/PD-1 blockade therapy, the expression level of TMB, tumor-infiltrating lymphocytes, PD-L1, and mismatch repair deficiency can all influence ICI therapy (Wang et al., 2019; Touat et al., 2020). But in the current phase III clinical trials, there was no screening of glioma patients for these elements, and this non-distinctive therapy may also account for the failure of these trials, which is a concern for future studies.

In response to the above-mentioned challenges in immunotherapy, this research presented a novel categorization of glioma on the basis of necroptosis. We observed that NI subgroup I presented higher NI and was more responsive to immunotherapy, which offered a novel way of selecting patients who were appropriate for immunotherapy. This study analyzed potential anti-glioma compounds in the “pRRophetic” package. For NI subgroup I, Gemcitabine, Bortezomib, Midostaurin, Lapatinib, Rapamycin, Tipifarnib, Etoposide, Embelin, Roscovitine, Docetaxel, Bexarotene, Pazopanib, and Dasatinib were reconsidered as the targeted drugs. In the case of NI subgroup II, Gefitinib, Axitinib, and Bosutinib were identified as the potential targeted drugs. These are the anti-tumor drugs approved by the FDA for future screening of anti-glioma drugs. Despite the absence of studies on drugs and immunotherapy, our analysis confirmed the validity of drug screening and the clinical translation of drug response to glioma treatment.

Ferroptosis, cuproptosis, and necroptosis are all important cell death modalities that play an important role in the tumor microenvironment (Shen et al., 2022; Xie et al., 2022; Zhang et al., 2023). It was found that ferroptosis could be involved in tumorigenesis, progression, and activation of different regulatory sites in the ferroptosis pathway and could promote tumor cell death. Related studies have shown that cuproptosis is involved in most mechanisms of tumorigenesis and metastasis and complicates tumor immune escape. Tumor cells undergo necrosis as a self-sacrificing strategy to create a favorable environment for their proliferation and metastasis, but necroptosis exerts tumor suppressive effects in most cases. Studying the relationship between cell death and the tumor microenvironment can further contribute to our understanding of how different cell death modalities affect tumor development and provide new ideas to inhibit tumor growth (Zou et al., 2022; Zou et al., 2023).

We reviewed some related literature and found that they all present systematic analysis of necroptosis mainly focusing on low-grade glioma and breast cancer (Xie et al., 2022; Zou et al., 2022), and there is little overall analysis of low-grade and high-grade gliomas. Moreover, previous studies on gliomas have directly performed model construction using key genes, and there is no integration of key genes’ enrichment scores to comprehensively evaluate the role of necroptosis-related genes in tumors.

We calculated necroptosis index (NI) using eight necroptosis related genes and found that it significantly responded to the prognosis of patients by NI in most tumors. We also studied its prognosis, immune environment, radiotherapy, and molecular-targeted therapy in glioma patients, and obtained relatively satisfactory results. We performed PCR validation of the expression of eight necroptosis genes in glioma samples, and this cross-corroboration of database and experiment further illustrates the reliability of our experiments.

Our analysis first correlated the role of necroptosis-related genes in pan-cancer and further found that necroptosis was significantly associated with the prognosis of glioma patients, and then further analyzed the close relationship between necroptosis and glioma. Such an analysis is more logical. However, our model has some drawbacks. First, we need to test the expression of these eight key genes in order to evaluate the prognosis of patients, and this associated cost may be high. Second, although the CGGA database has a considerable number of samples to validate the conclusions, we need to develop the sample number of our hospital in the future. Third, due to the very limited number of patients receiving immunotherapy and our study being supported by public databases, the relationship between immunotherapy and NI subgroups needs to be investigated in future immunotherapy cohorts.

## Conclusion

In summary, there remains a great potential for immunotherapy in glioma. Screening patients who may be more suitable for immunotherapy is essential. In this research, we classified patients into two distinct subtypes according to NI of glioma, and predicted sensitivity of patients in two subgroups to immunotherapy, offering a method for screening suitable patients for immunotherapy. Our study also identified predictors of NI subgroups, which makes it clinically feasible to translate NI.

## Data Availability

The original contributions presented in the study are included in the article/Supplementary Material, further inquiries can be directed to the corresponding authors.

## Abbreviations

DEGs, differentially expressed genes; GBM, glioblastoma; LGG, low grade glioma; NI, necroptosis index; ROS, reactive oxygen species; ssGSEA, the single sample gene set enrichment analysis; SCNA, somatic copy number alterations; SNP, single nucleotide polymorphisms; NRGs, necroptosis regulator genes.
