# Supplementary material for: Systematic analysis of the necroptosis index in pan-cancer and classification in discriminating the prognosis and immunotherapy responses of 1716 glioma patients
Source: Front Pharmacol. 2023 Jun 7;14:1170240. doi: 10.3389/fphar.2023.1170240 (PMC10282546; doi:10.3389/fphar.2023.1170240)
Supplement: Supplementary file 2 [file DataSheet1.docx]

# Supplementary Materials

## Supplementary figures

**Figure S1.** Kaplan-Meier Analysis of Overall Survival according to the FPI among Cancers.


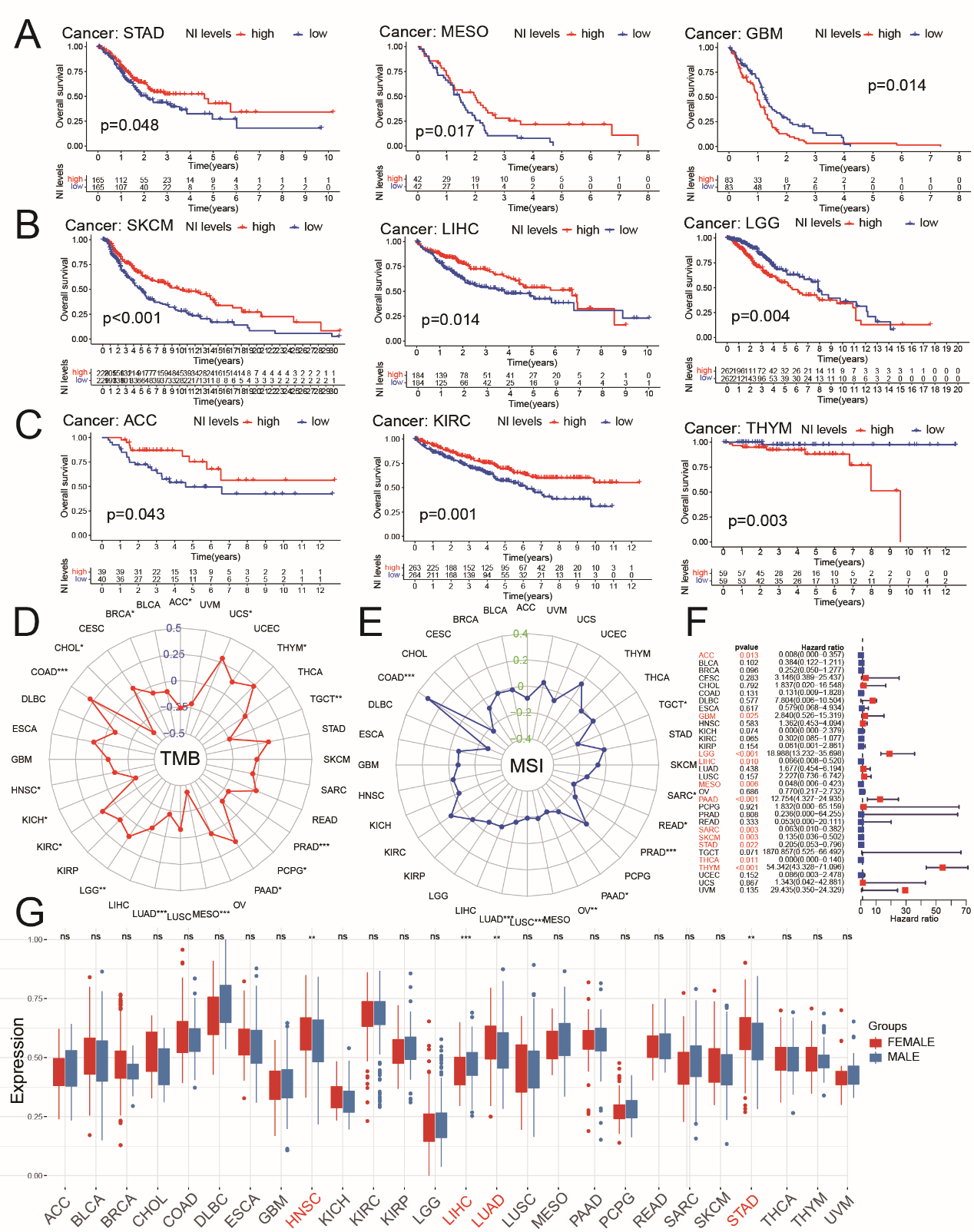


**(A-C)** Kaplan-Meier Analysis of Overall Survival according to the NI among Cancers. **(D)** Radar chart of the correlation between hub genes and TMB. **(E)** Radar chart of the correlation between hub genes and MSI. **(F)** Forest showed the prognostic value of NI in pan-cancer. **(G)** The differential expression of NI between gender in pancancer.

**Figure S2.** The panorama of gene and expression alteration of necroptosis regulators in glioma.


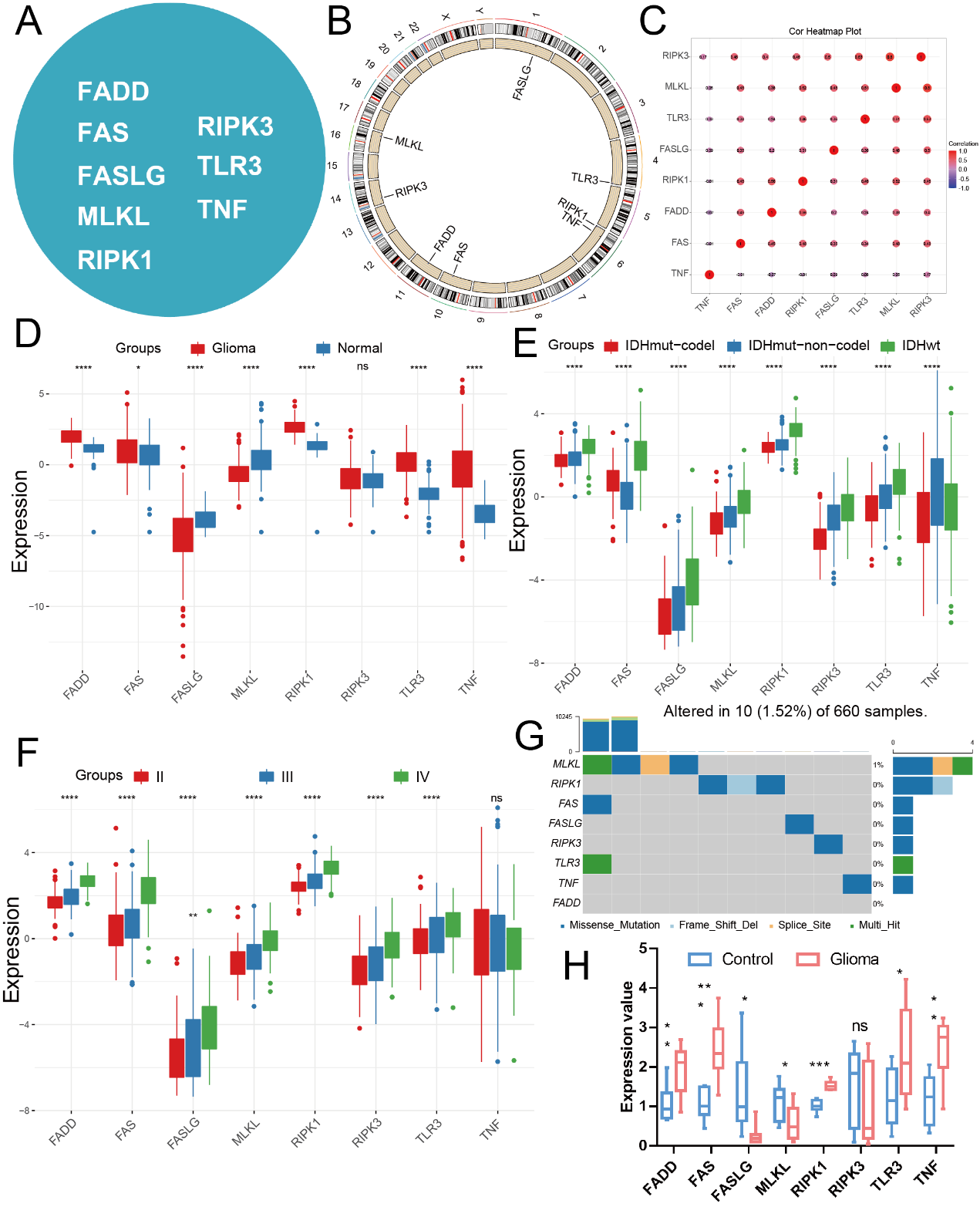


**(A)** The overview of necroptosis regulators. **(B)** The position of CNV variation of necroptosis regulators on twenty-three chromosomes from the TCGA-glioma cohort. **(C)** Spearman correlation analysis of the necroptosis regulators. **(D)** The expression of necroptosis regulators between molecular subtypes. **(E)** The expression of necroptosis regulators between normal and glioma samples. **(F)** The expression of necroptosis regulators among grades. **(G)** The mutation frequency of necroptosis regulators in glioma. **(H)** PCR validation of necroptosis genes.

**Figure S3.** The relationship between NI and immunity.


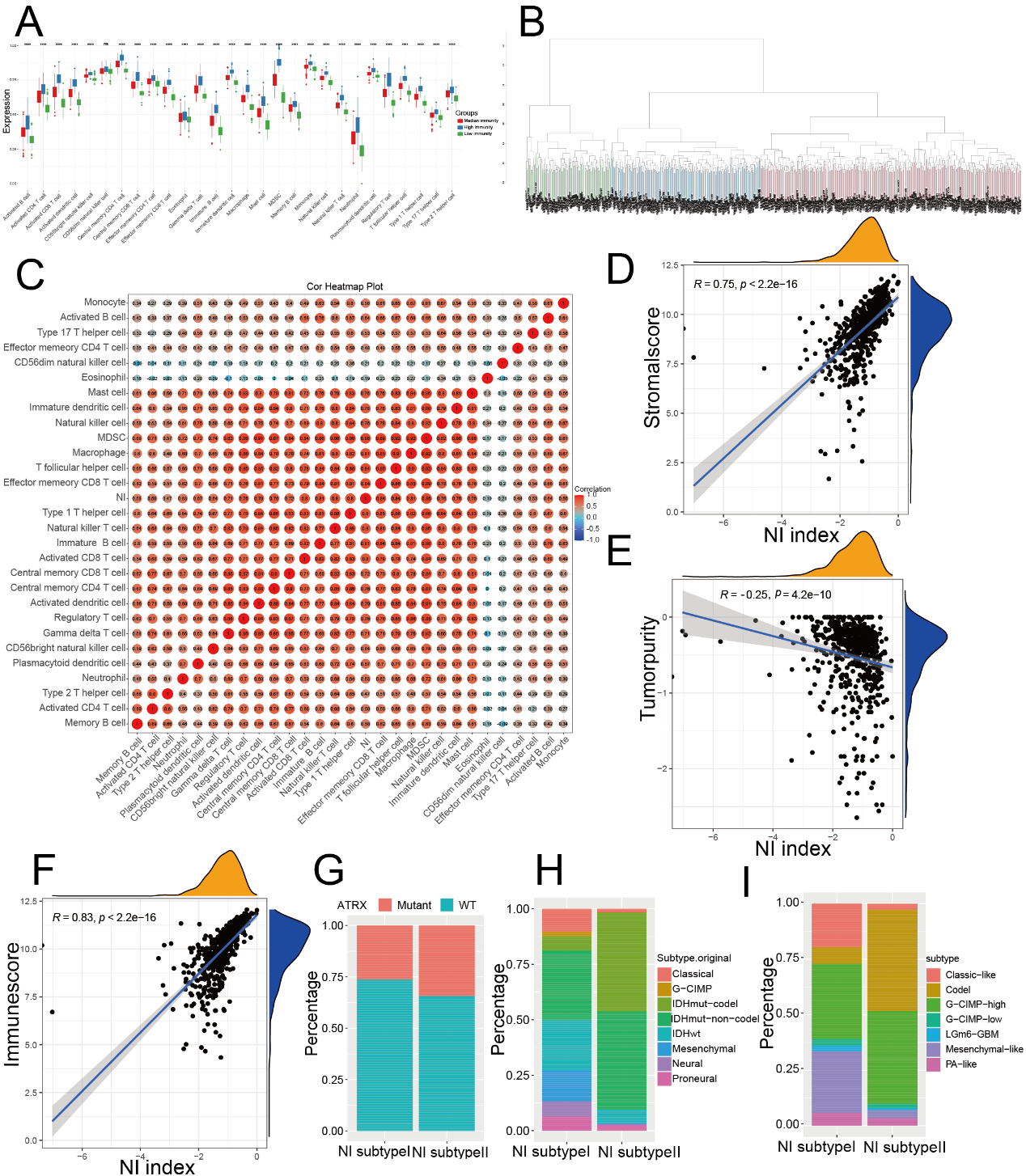


**(A)**The relationship between 22 immune cells and three immune subtypes. **(B)** The Percentage of patients in the three immune subtypes. **(C)** Correlation of NI with 22 types of immune cells. **(D,E)** Correlation of NI with ESTIMATETscore. **(G-I)** The proportion of ATRX mutations, classically typed in the NI subtypes.

**Figure S4.** The proportion of the clinical traits in the two NI subtypes.
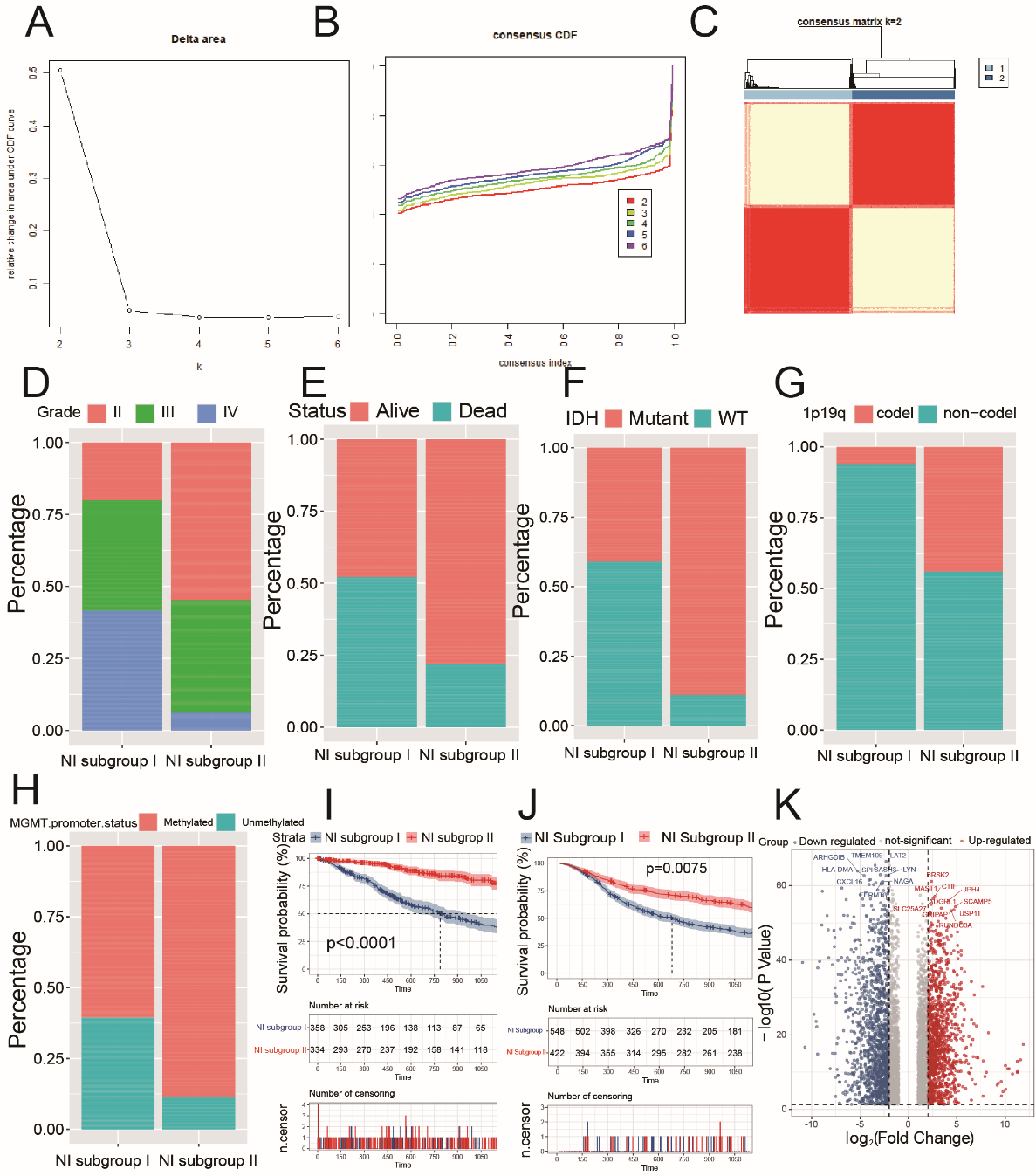


**(A,B)** Consensus Cumulative Distribution Function (CDF) Plot for immune-related genes for glioma. **(C)** Color-coded heat maps corresponding to the consensus matrices. **(D-G)** The percentage of grades, OS, IDH mutation, 1p19q, and MGMT methylated in two NI subtypes. **(I)** KM curves to calculate the survival of two NI subtypes in the TCGA. **(J)** KM curves to calculate the survival of two NI subtypes in the CGGA. **(K)** Volcano map displaying the differential genes of two NI subtypes.

**Figure S5.** Comparisons of clinicalpathological and somatic variations between NI Subtype I and II.


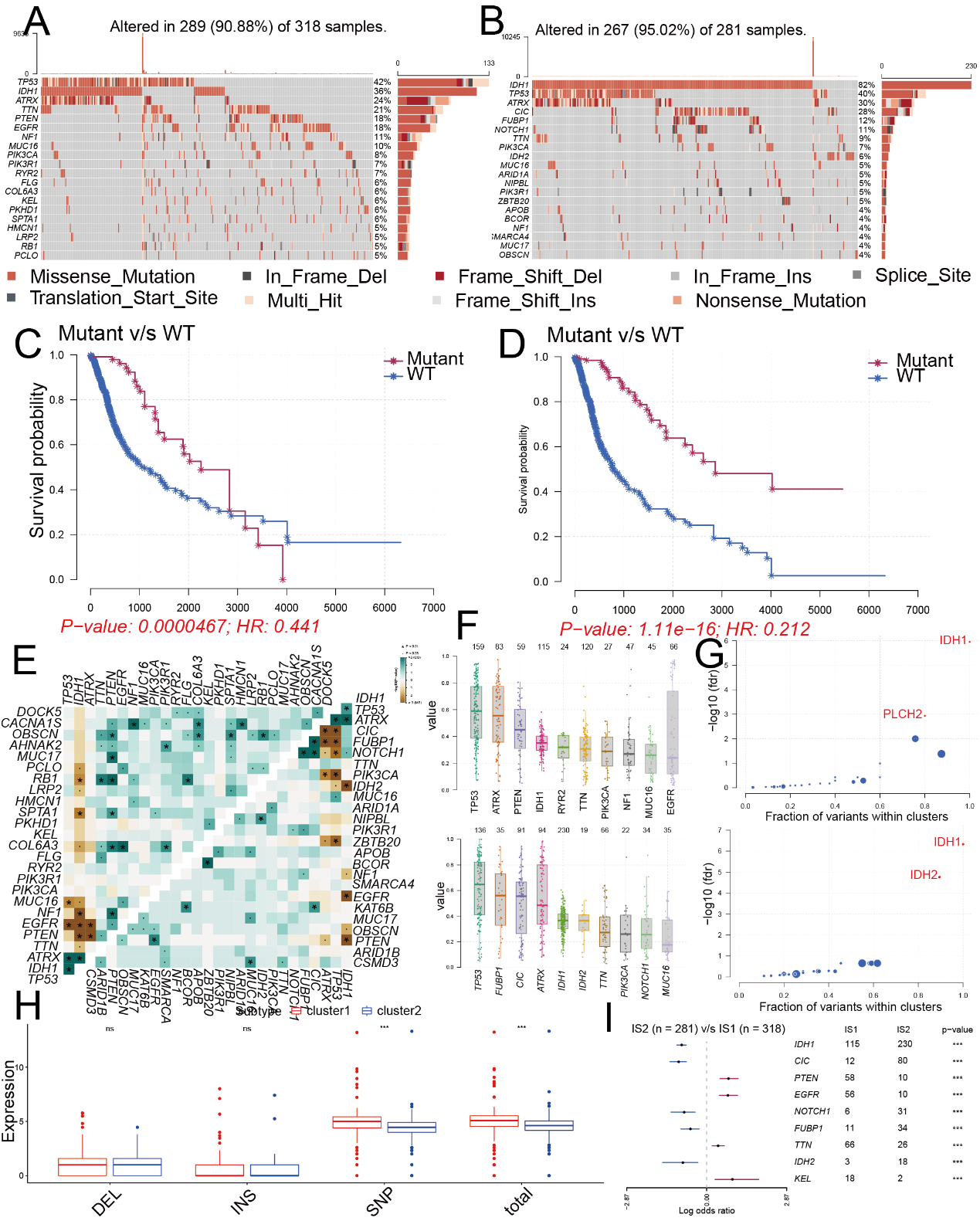


**(A,B)** Waterfall plots showed the top 10 mutated in NI Subtype I and II. **(C,D)** Kaplan-Meier curves show the independent relevance between overall survival time and STK11 mutation in two subtypes. **(E)** Heatmap showing the mutual co-occurrence and exclusive mutations within the top 25 frequently mutated genes. **(F)** Variant Allele Frequency expression of the top 10 genes of the two subtypes. **(G)**Distribution of tumor driver genes in two subgroups. **(H)** Each mutation type is classified by effects, INDEL, and INS, SNP. **(I)** Forest plot displays the top 10 most significantly differentially mutated genes between four cohorts.

**Figure S6.** Identification and validation of the NI subgroups predictor.


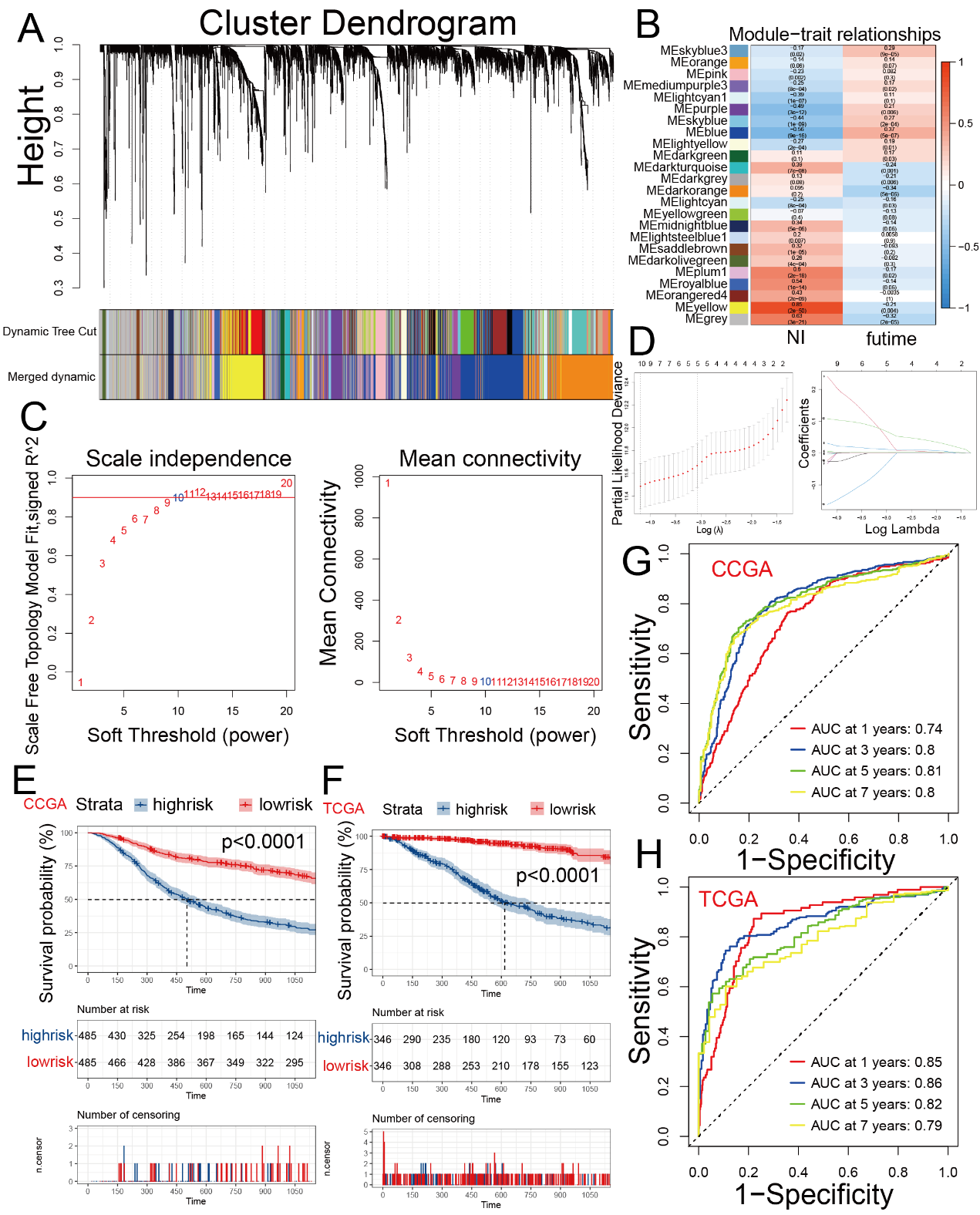


(A) The co-expressed genes identified in the modules by hierarchical clustering dendrogram. (B) Relevance of gene modules to NI and OS. (C) WGCNA process of NI subgroups-related DEGs with a soft threshold β = 10. (D) Ten-time cross-validation for tuning parameter selection in the TCGA cohort. (E,F) KM curve plot of OS for patients in high and lowrisk subgroups in TCGA and CGGA cohort. (G,H) the timeROC curve to evaluate the prognostic model in TCGA and CGGA cohort.

**Figure S7.** Estimated drug sensitivity in patients with two NI subtypes.


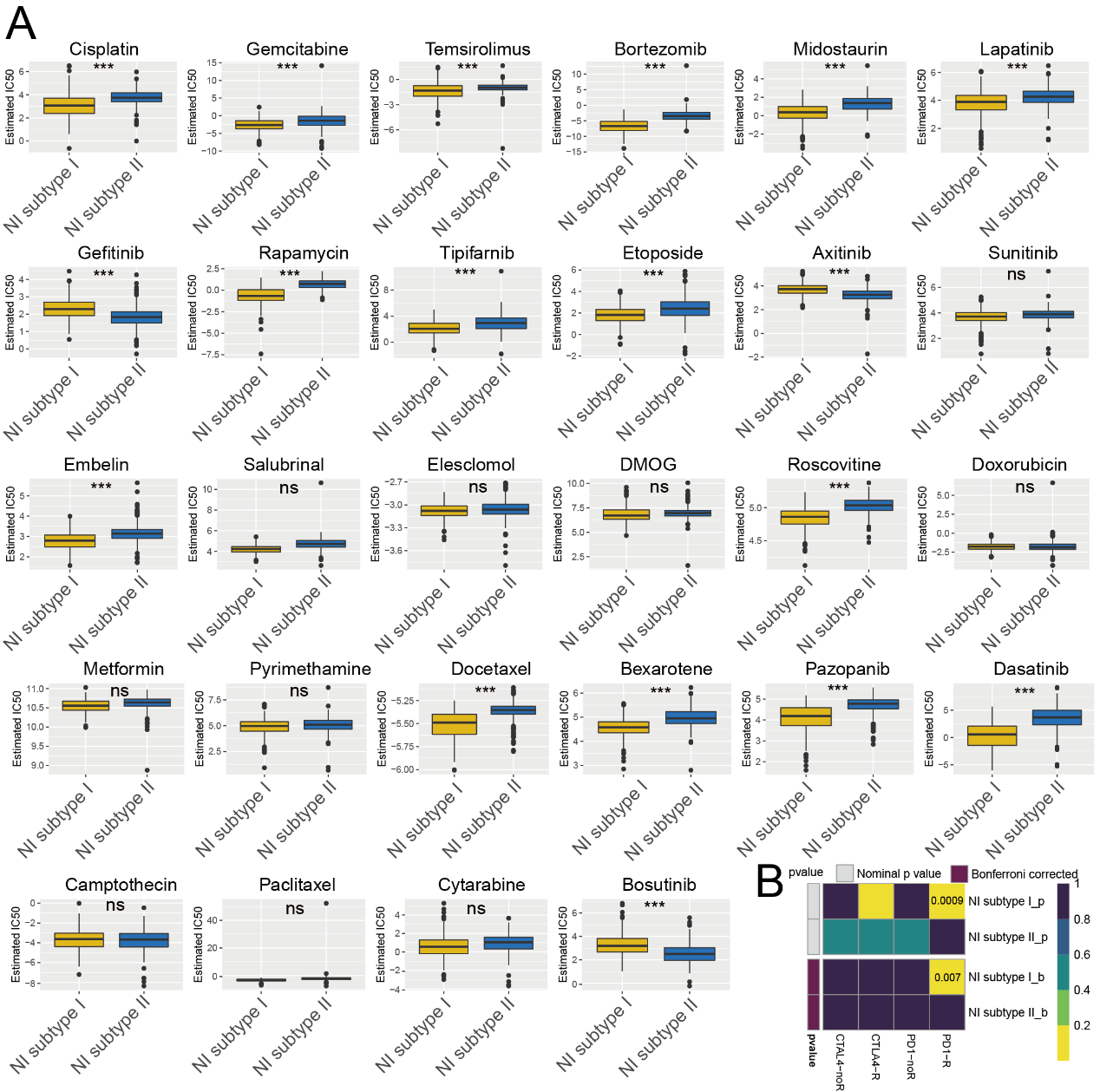


**(A)** The Chemotherapeutic reaction of PIPM for 30 prevalent chemotherapy drugs.**(B)** Comparison of the effectiveness of NI subtypes in predicting ICB responsiveness.
